# Characterization of the oral microbiota and the relationship of the oral microbiota with the dental and periodontal status in children and adolescents with nonsyndromic cleft lip and palate. Systematic literature review and meta-analysis

**DOI:** 10.1007/s00784-024-05624-3

**Published:** 2024-04-08

**Authors:** Francina Escobar-Arregocés, Mayra-Alexandra Eras, Andrea Bustos, Angela Suárez-Castillo, Dabeiba-Adriana García-Robayo, Maria del Pilar Bernal

**Affiliations:** 1 Center of Dental Research, Member of the interdisciplinary team for the care of patients with CLP, Faculty of Dentistry, Pontificia Universidad Javeriana, Bogotá, DC Colombia; 2 Pediatric Dentistry, Faculty of Dentistry, Pontificia Universidad Javeriana, Bogotá, DC Colombia; 3 Public Health, Faculty of Dentistry, Pontificia Universidad Javeriana, Bogotá, DC Colombia; 4 Biological Science, Center of Dental Research, Faculty of Dentistry, Pontificia Universidad Javeriana, Bogotá, DC Colombia; 5 Pediatric Stomatology, Member of the interdisciplinary team for the care of patients with CLP, Faculty of Dentistry, Pontificia Universidad Javeriana, Bogotá, DC Colombia

**Keywords:** Cleft lip and palate, Microbiology, Biofilm, Dental caries, Periodontal disease

## Abstract

**Objective:**

To identify the characteristics of the oral microbiota and the relationship of the dental caries and periodontal status in patients aged 0 to 18 years with non-syndromic cleft lip and palate (CLP).

**Materials and methods:**

A systematic review of the literature was carried out. Five databases were consulted, including publications in English, Spanish and Portuguese. The evaluations of the quality of the observational studies and the experimental studies were carried out with the Newcastle–Ottawa scale and CONSORT guidelines, respectively. The risk of bias of the studies was determined using Rev Manager 5.4, and 5 publications were meta-analyzed.

**Results:**

The cariogenic microbiota of children and adolescents with cleft lip and palate was similar to that of children without clefts, although with higher counts of Streptococcus mutans and Lactobacillus spp. The periodontopathogenic microbiota was related to the presence of Campylobacter spp, Fusobacterium spp, Fusobacterium nucleatum, Prevotella intermedia/nigrescens, Parvimonas micra and Porphyromonas gingivalis, considered microorganisms with high pathogenic capacity. Heterogeneity was shown in relation to the microbiota and the type of fissure, presenting numerous microorganisms associated with the pre- and post-surgical condition (cheilorrhaphy and palatorrhaphy) such as Staphylococcus aureus, Streptococcus beta hemolyticus, Klebsiella pneumoniae and Klebsiella oxytoca, Moraxella catarrhalis, Candida spp, Candida albicans, Candida krusei and Candida tropicalis. The meta-analysis revealed that patients with cleft lip and palate were 2.03 times more likely to have caries than the control group (*p*<0.005).

**Conclusion:**

In the microbiota, there was a great diversity of microorganisms that can vary according to the type of fissure and surgical interventions predisposing patients to a greater probability of dental caries, it is important to take into account the technique used to describe the oral microbiota in order to be able to compare the different studies.

**Clinical relevance:**

Studying the microbiota and the relationship of dental caries and periodontal status in children and adolescents with cleft lip and palate can facilitate the comprehensive care of patients with these conditions.

## Introduction

Cleft lip and palate (CLP) is one of the most frequent congenital craniofacial malformations and originates between the fourth and sixth week of intrauterine life [1, 2]. The World Health Organization (WHO) reports a worldwide yearly incidence of CLP of 1 in every 700 live births [3]. In South America, the prevalence has been reported to be 1 in 800 live births, and in Colombia, according to the fourth national study of oral health in Colombia (ENSAB IV 2013-2014), the prevalence is 1 in 700 (0.07%) [3].

This malformation develops as an anatomical defect in the fissured area, contributing to problems with phonation, chewing, and swallowing, malposition and dental alterations in shape, number and structure; this discontinuity of the tissues of the oral and nasal cavities contributes to the formation of unbalanced bacterial ecosystems [1, 4–6]. In the first months of life, surgical interventions are generally performed to repair the defect through primary closure of the fissure, for which the literature has shown [3] a higher prevalence of gram-negative microorganisms before surgery and a higher frequency of gram-positive microorganisms after surgery, related to the surgical closure of the nasopharyngeal space. Furthermore, the risk of early colonization of microorganisms increases significantly in children with CLP [3].

The microbiota in patients with CLP has been extensively investigated, and it has been suggested that children and adolescents with CLP may have elevated levels of *Streptococcus mutans, Candida* spp*.* and *Lactobacillus* spp*.* in saliva before and after cheilorrhaphy and palatorrhaphy [3, 7]. In some CLP patients, beta hemolytic *Streptococcus* and *Staphylococcus aureus* have been isolated from the nasal cavity [2], and the presence of *Candida* spp. has been shown to be related to the immunosuppression present at the time of birth [8].

Nonsyndromic cleft lip and palate (NSCLP) predisposes patients to the formation of retentive areas of biofilm, and as sequelae of surgical intervention, recurrent oronasal fistulas, wound dehiscences and scarring flanges appear, generating retention zones, which together cause different pH values, local oxygen concentrations, redox states, ionic compositions, buffering capacity and mechanical interactions, which, after the accumulation of food and oral and nasal fluids, create an environment conducive to the growth of various bacterial groups [1, 7]. Changes in the microbiota can produce alterations in the ecological balance, causing environmental disturbances that lead to a predominance of harmful microorganisms that contribute to the pathogenesis of oral diseases such as tooth decay and periodontal disease, among other pathologies [1, 9].

In this sense, different authors mention that the oral microbiota is different in children who present this condition than in children who do not present it [10, 11]. However, a consensus has not yet been established regarding the characterization of the oral microbiota in this population and whether it differs from the microbiota of the population without NSCLP. Therefore, the aim of this study was to identify the characteristics of the oral microbiota and the relationship of the oral microbiota with dental and periodontal status in patients aged 0 to 18 years with NSCLP.

## Materials and methods

A systematic review of the literature was performed in accordance with the PRISMA 12 guidelines [12]. The following question was posed under the PICO structure: P Children and adolescents aged 0 to 18 years with non-syndromic cleft lip and palate; I Oral microbiota; C Children and adolescents aged 0 to 18 years without non-syndromic cleft lip and palate; O Dental and periodontal status.

What are the characteristics of the oral microbiota in children and adolescents aged 0 to 18 years with non-syndromic cleft lip and palate and the relationship of the oral microbiota with dental and periodontal status?

### Eligibility criteria

Studies that evaluated the oral microbiota through analytical observational designs and clinical trials were included. Articles that included patients had received antimicrobial therapy were excluded.

### Search strategy

Electronic searches were carried out in Medline-PubMed, Embase-Elsevier, EBSCO, Scopus, and Web of Science, in addition to a gray literature search in Google Scholar. The search was restricted to articles in English, Spanish and Portuguese published from January 1, 1985, to June 30, 2020. Search descriptors were used in controlled and uncontrolled language related to *cleft lip, cleft palate, cleft lip and palate no syndromic, microbiology, biofilm, dental caries, periodontal diseases, dental caries susceptibility* and uncontrolled language such as *child, children, teenagers, oral microbiota, oral microbiome, periodontal state,* and *dental state.* Different combinations of terms were used in the search strategies through the Boolean operators AND, OR and NOT; the search strategies were tailored to the particularities of each database.

### Methodological evaluation of the publications

Assessment of the methodological quality of the publications was carried out through the Newcastle–Ottawa scale (NOS) [13] for analytical observational studies and the Consort checklist for clinical trial.

### Statistical and analytical aspects

The search and extraction of the information was carried out independently by 4 reviewers. Once the data were extracted from the articles, they were analyzed using *Review Manager 5.4* software.

To calculate the effect size, the articles that included ORs with the 95% CIs were taken into account, as well as the raw data for the studies with an analytical observational design. Means were analyzed to determine differences; heterogeneity was assessed using the Q statistical method based on v2 and I2, with significance indicated by *P* < 0.05.

## Results

**Fig. 1:**
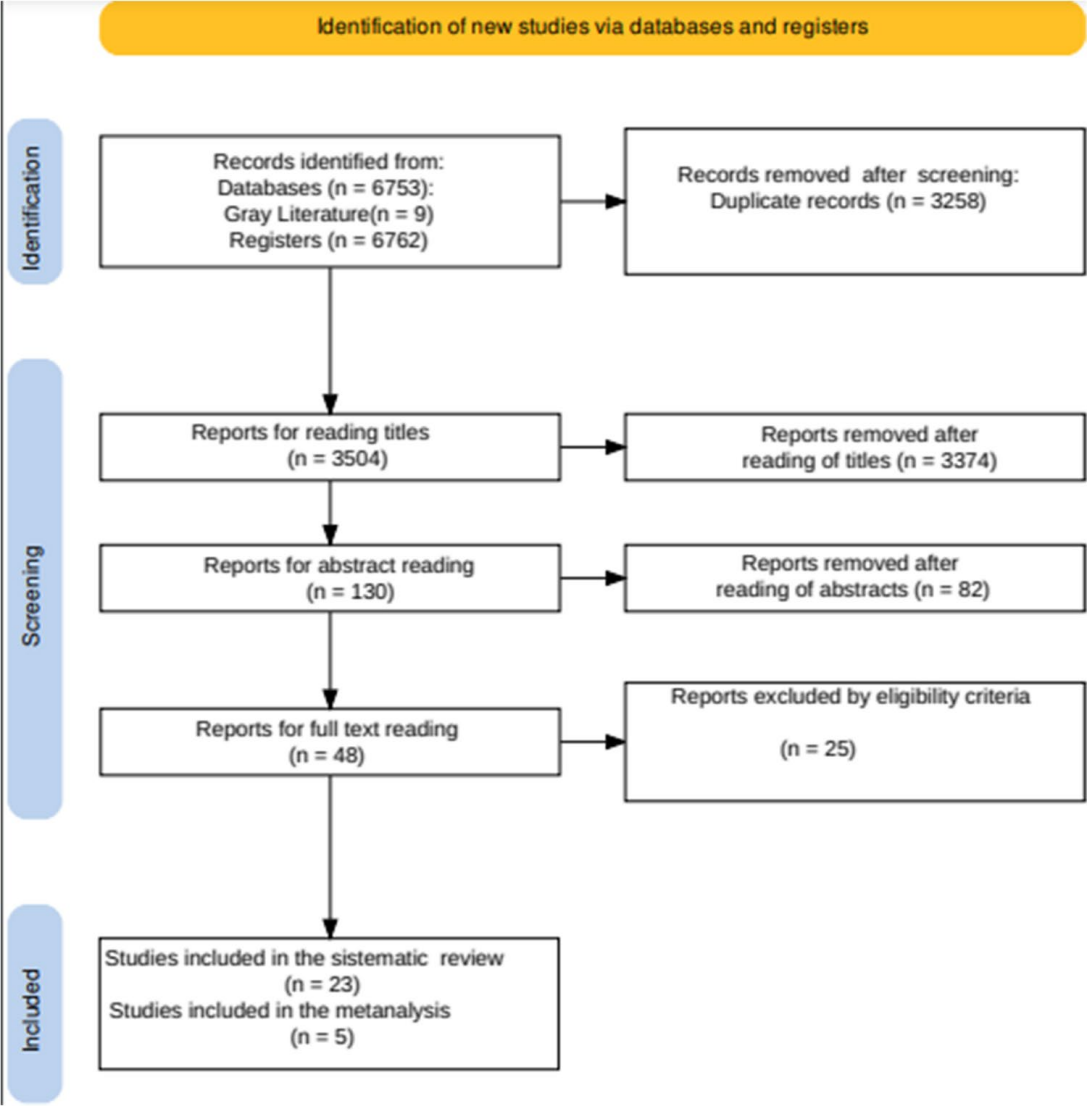
Flow diagram. Selection of articles for the systematic literature review

The flowchart for article inclusion is shown in Fig. 1.

The essential information for each study is summarized in Table 1

**Table 1:** Essential information from the studies

Author- Year	Study design	Study population age(years)	Study group	Control group	Analysis	Results	Conclusions
Ahluwalia M, 2004. London [14]	Analytical prevalence	6-16	81	41	Microbiological culture Colony forming units (CFU/mL) Saliva samples	• Salivary levels of *Streptococcus mutans, Lactobacillus* spp and yeasts were significantly higher (*p* < 0.001) in children with cleft palate than in the control group. • Compared to children in the control group, children with cleft palate had significantly higher median DMFT 1 and median DFT 2 scores (*p* < 0.001). • Compared to children in the control group, children with cleft palate had a significantly higher gingival index (*p* < 0.001).	There was no clarity regarding the higher frequency of cavities in children with cleft palate.
Lucas V, 2000. London [15]	Analytical prevalence	3-15	60	60	Microbiological culture Isolation frequency Dental plaque samples	• *Streptococcus mutans*, median 1.58 between the distal proximal site and the remote site of the maxillary cleft (*p* < 0.01) and median 2.13 between the contralateral anterior site and the remote site of the maxillary cleft (*p* < 0.04). • *Lactobacillus* spp*.,* median of 0.46 between the distal proximal site, median of 0.23 in the anterior contralateral site and median of 0.18 in the remote site of the maxillary cleft (*p* > 0.05).	There were no significant differences between the children with CLP and the control group for the DMFT index, dental plaque and gingivitis in the deciduous and permanent dentition.
Durhan M, 2018. Turkey [16]	Observational descriptive longitudinal prospective	0-3 years (newborns)	21 CLP	13	Microbiological culture CFU/mL Saliva samples	• *Streptococcus mutans,* present in the saliva samples of 10% of babies with CLP at birth. • Compared to the control group, 7 babies with CLP presented higher *Lactobacillus* spp infection at birth (*p* = 0.029), and 12 babies presented with a fissure after the eruption of the first deciduous tooth (*p* = 0.030). • There was no statistically significant relationship between initial caries and the presence of microorganisms.	Patients with CLP could be considered a group with a higher risk of caries.
Sundell A, 2018. Sweden [6]	Cross section	5	80 fissures	144	DNA–DNA hybridization Bacterial count Saliva samples	• Compared to the control group, children with fissures had a higher prevalence of caries (18% vs.36%; *p* < 0.05). • *Streptococcus mitis* (73% and 90%), *Streptococcus gordonii* (61% and 82%), *Fusobacterium nucleatum* (55% and 75%), and *Rothia dentocariosa* (50% and 58%) were frequently detected in the study and control groups, respectively. • *Bifidobacterium dentium* (6%), *Fusobacterium nucleatum* (55%), *Streptococcus gordonii* (61%), *Streptococcus mitis* (73%), *Streptococcus salivarius* (35%), and *Veillonella parvula* (6%) were less frequent in patients with CLP (*p* < 0.05).	Children with fissures had a higher prevalence of caries.
Cocco JF, 2010. United States [17]	Analytical prevalence	6-12 months	35 primary cleft lip repair, 44 undergoing palatoplasty	---	Microbiological culture Bacterial count Nasal, sublingual and oropharyngeal samples	• *Klebsiella pneumoniae* was present in the oropharynx of 56% of CLP patients with primary lip repair. • Increased *Staphylococcus aureus* colonization in 34% of patients with isolated cleft palate before the operation (*p* = 0.298). • Methicillin-resistant *Staphylococcus aureus* was present in 2.3% of patients at 6 months and increased to 4.5% at 12 months after palatoplasty. • *Klebsiella pneumoniae* and *Enterobacter cloacae* decreased significantly, by 14% and 4.5%, respectively, in the oropharynx 12 months after the closure of the palatal cleft (*p* < 0.05). • The major complication was palatal dehiscence and was directly related to group A beta-hemolytic *Streptococcus* (*Streptococcus pyogenes*) infection (no source data).	The colonization of *Streptococcus pyogenes* was associated with a high risk of wound dehiscence.
Rawashdeh MA, 2011. Jordan [8]	Analytical cross section	5-17	60 with fissures	60	Fungi culture Identification of *Candida* species Tongue, nasal mucosa and palatal samples	• Colonization by *Candida* spp*.* was greater in patients with bilateral CLP (77.7%) than in patients with unilateral CLP and cleft palate (57.1%). • There was a statistically significant difference in the rate of colonization by *Candida* spp, between patients with cleft palate who underwent 3 surgical interventions (78.2%) and those who underwent 1 surgical intervention (40%) (*p* = 0.01). • Cleft patients and the control group showed a gingival and plaque index of 1. • Cleft patients had significantly higher DMFT (3.3) and ceod (2.93) scores than did the control group (*p* = 0.0001 and *p* = 0.015). • Type of cleft and number of surgical interventions did not influence the gingival index, plaque index, of DMFT and ceod scores.	The patients with cleft presented greater colonization by oral *Candida* than did the control group; this varied with age, type of cleft and number of surgical interventions.
Tuna E, 2008. Turkey [18]	Correlation cross section	-----------	Complete unilateral CLP and complete bilateral CLP	---------	Microbiological culture CFU/mL Salivary and nasal samples	• *Staphylococcus aureus* was present in 53.1% of saliva samples and 40.6% of nasal samples in fissured patients.	Children with oronasal fistula had a higher count of *Staphylococcus aureus* in saliva than did children without fistula.
Arief E, 2005. Malaysia [19]	Before-after intervention	3-39 months	15 CLP	22	Microbiological culture CFU/mL Salivary samples	• *Streptococcus mitis,* preoperative 6%, postoperative 0%; *Streptococcus biovar* preoperative 25%, postoperative 28.7%; *Streptococcus salivarius* preoperative 21%, postoperative 23.8% and *Streptococcus oralis,* preoperative 21%, postoperative 28.7%. • *Staphylococcus aureus* was more frequent in patients with CLP in the preoperative phase (47.4%), with 0% in the postoperative phase; the difference was statistically significant (*p* < 0.05).	Patients with CLP presented greater colonization of microorganisms in the oral cavity. Colonization decreased after lip and palate repair.
Cheng Ll, 2007. Australia [20]	Nonrandomized clinical trial	12-17	Two study groups with and without CLP and with orthodontic treatment	Two control groups with and without palatal CLP without orthodontic treatment	CRT bacteria test CFU/mL Salivary samples	• *Lactobacillus* spp, statistically significant differences in the percentages of subjects with ≥ 10 ^5^ CFU/mL between the group with treatment without cleft (76.7%), the group with cleft treatment (73.3%), the control group without cleft (46.7%) and the control group with cleft (40%) under treatment with fixed appliances. • Highest and lowest percentages of subjects with ≥ 10 ^5^ CFU/mL of *Streptococcus mutans* were in the group without cleft with treatment (86.7%) and the group with cleft treatment (60%).	Children with CLP and fixed orthodontics had more favorable microbiological and salivary profiles for the development of caries.
Funahashi K, 2019. Japan [21]	Series of cases operated	7-15	6 CLP	4	Bacterial identification 16S rRNA OTU (operational taxonomic units) Supragingival plaque samples	• The most predominant genera were *Actinomyces* spp *(*14.0% in the CLP group and 10.6% in the control group), *Corynebacterium matruchotii* and *Leptotrichia hofstadii, (*4.9 ± 0.80% in the cleft lip and palate group and 4.3 ± 4, 1% in the control group). • Fifteen taxa were identified in the group with CLP and 3 taxa in the control group: *Lactobacillus rhamnosus, Lactobacillus fermentum, Streptococcus salivarius, Prevotella pallens, Aggregatibacter aphrophilus, Streptococcus gordonii, Streptococcus cristatus, Prevotella pleuritidis, Capnocytophaga gingivalis, Prevotella marshii, Prevotella micans, Streptococcus anginosus* spp*., Catonella morbi* and *Selenomonas artemidis* in the CLP group and *Prevotella loescheii, Stomatobaculum longum,* and *Streptococcus sanguinis* in the control group. • *Leptotrichia* spp*.* and *Neisseria* spp. were the most predominant genera on average in the CLP group and in the control group (13.2% and 16.8%), respectively. • Gingival index score was higher than 0 for 2 subjects with CLP and 1 control subject. • DMFT score was greater than 0 for 3 subjects with CLP and 1 control subject.	Functional dysbiosis in the oral microbiota in patients with CLP changed unpredictably and could be associated with cariogenicity.
Liu L, 2016. United States [9]	Before and after	8-16	28 CLP	---	Bacterial identification 16S rRNA OTU (operational taxonomic units) Saliva samples	• The most abundant phyla were *Firmicutes* spp (mean of 38.1% in the inflammation group and mean of 39.3% in the noninflammation group), *Proteobacteria* spp (31.2% in the inflammation group and 32.9% in the noninflammatory group), *Bacteroidetes* spp (17.8% in the inflammation group and 16.1% in the noninflammation group), *Actinobacteria* spp (7.4% in the groups with and without inflammation), and *Fusobacteria* spp (3.6% in the inflammation group and 2.5% in the noninflammation group). These five predominant phyla constituted 98.1% of the total microbiota in the inflammation group and 98.2% of the total microbiota in the noninflammation group. • Inflammation-related OTUs were *Tannerella* spp, *Porphyromonas* spp, *Gemella* spp, *Moraxella* spp, *Prevotella nigrescens* and *Prevotella intermedia*. • Related OTUs without the presence of inflammation were *Lautropia* spp, *Neisseria* spp, *Capnocytophaga* spp, *Veillonella dispar*, *Veillonella parvula* and *Prevotella melaninogenica*. • OTUs corresponding to *Streptococcus* spp*.* and *Prevotella* spp*.* were present in both groups. • Enriched OTUs in subjects without inflammation showed a positive correlation (Spearman's correlation coefficient t > 0.4, *P* < 0.05). • Inflammation-enriched OTUs included *Tannerella* spp., *Porphyromonas* spp*.*, *Gemella* spp., *Moraxella* spp*.*, and *Prevotella nigrescens*.	The salivary microbial composition varied significantly by surgical procedures between subjects with CLP with postoperative inflammation and without inflammation.
Hassani H, 2020. United States [22]	Nonrandomized clinical trial	2-11 months	23 with nasoalveolar molding, 8 without nasoalveolar molding	30	CRT bacteria test Colony count Saliva samples	• *Streptococcus mutans* colony count, median 63; *Lactobacillus* spp. colony count, median 464.1; *Streptococcus mutans* caries risk test, median 3; and *Lactobacillus* risk test, median 2.6, were significantly different (*p* < 0.0001) in patients with CLP with a nasoalveolar molding.	There was a high bacterial count and high risk of caries in the group that used nasoalveolar molding.
Zhang M, 2016. Amsterdam [2]	Nonrandomized clinical trial	1 year	10	10	PCR-DGGE Bacterial composition Saliva and nasal samples	• *Lautropia* spp. (40%) and *Bacillus* spp. (10%) were significantly less present in the saliva samples of the group with complete cleft palate (*P* = 0.029). • *Dolosigranulum* spp*.* (100%) were more predominant in the nasal cavity of the control group and less frequent (50%) in the group with complete cleft palate (*P* = 0.016). • *Bacillus* spp. (10%) was present in the nasal samples of the group with complete cleft palate (*P* =.029). • *Streptococcus* spp. (80%) were more prevalent in nasal swabs from children with CLP than in those from children in the control group (*p* = 0.012).	The altered ecological ecosystem in the oral and nasal microbiome of children with cleft palate is presented as a consequence of abnormal communication between the two cavities.
Loveren C, 1998. The Netherlands [23]	Analytical prevalence	12 to 18 months	21 who use PNAM, 28 with cleft lip and palate	35	Microbiological cultures CFU/μL Saliva and dental plaque samples	• At 18 months of age, there was no difference in the prevalence of *Streptococcus mutans* (20%) (chi-square *P* = 0.4). • All children with an acrylic plate colonized with *Streptococcus mutans,* at the age of 9 and 13 months, were colonized with *Lactobacillus* spp. • In 6-month-old children, 80 strains of *Lactobacillus* spp. were identified Three of these strains were identified as *Lactobacillus jensenii*. • Presence of *Lactobacillus* spp., with the possibility of 8 (95% CI 1.5-43.2; *p* < 0.05) stemming from presurgical orthopedics.	The presence of Streptococcus mutans in the saliva of children with oral fissure was associated with the consumption of snacks and with the presence of Lactobacillus spp.
Bokhout B, 1996. The Netherlands [24]	Analytical prevalence	62 children between 18 months and their mothers	---	------	Microbiological cultures CFU/mL Saliva and dental plaque samples	• *Streptococcus mutans* in saliva (45.2%) and in plaque (48.4%); *Lactobacillus* spp, 16.1% in saliva and 8.1% in plate. • Cleft lip: *Streptococcus mutans* in saliva (38.5%) and in teeth (23.1%); *Lactobacillus* spp in saliva and teeth (0%). • Alveolar cleft lip: *Streptococcus mutans* in saliva (50%), in teeth (40%); *Lactobacillus* spp in saliva (20%) and in teeth (0%). • Unilateral cleft lip and palate: *Streptococcus mutans,* in saliva (44.4%) and in teeth (61.1%); *Lactobacillus* spp., in saliva (33.3%) and in teeth (22.2%). • Bilateral cleft lip and palate: *Streptococcus mutans*, in saliva (57.1%) and in teeth (71.4%); *Lactobacillus* spp., in saliva (14.3%) and in teeth (14.3%). • Palatal cleft: *Streptococcus mutans*, in saliva (42.9%) and in teeth (50%); *Lactobacillus* spp, in saliva (7.1%) and in teeth (0%). • *Lactobacillus* spp, in saliva OR 4.7 (95% CI, 1.00-22.45). • *Lactobacillus* spp in the saliva of the children depended on the presurgical orthopedics, OR 4.8 (95% CI, 1.10-20.92).	Children with fissures had an increased risk of being infected with Streptococcus mutans and Lactobacillus spp. at a very early age, and colonization indicated a high risk of caries in the primary dentition.
Perdikogianni H, 2009. Greece [11]	Analytical cross section	4-18	41	41	Microbiological culture CFU/mL Subgingival plaque samples	• Gram-positive facultative anaerobic cocci, *Gemella haemolysans, Streptococcus* spp., *7.9 x 10*^*6*^ in fissured children's molars and *8.7 X 10*^*7*^ in control molars*;* gram-positive facultative anaerobic bacilli, *Actinomyces* spp*., Lactobacillus* spp., and *Rothia dentocariosa, 5.2 X 10*^*6*^ in fissured child molars and 4.3 X 10^6^ in control molars; facultative gram-negative anaerobic bacilli *Capnocytophaga* spp., *Eikenella corrodens,* and *Haemophilus* spp., 3.8 X 10^6^ in fissured children's molars and 3.0 X 10^7^ in control molars; gram-negative anaerobic bacilli, *Bacteroides* spp., *Bilophila wadsworthia, Campylobacter* spp., *Wolinella* spp., *Fusobacterium* spp*, Porphyromonas gingivalis, Prevotella intermedia / nigrescens, Prevotella loeschii, Prevotella melaninogenica, Prevotella melaninogenica,* and *Prevotella* spp., 5.5X 10^6^ in fissured child molars and 4.0 X 10^5^ in control molars (*p* < 0.005). • There was a significant difference in the probing depth of the upper anterior teeth between the study group and the control group (*p* < 0.05). • The teeth close to the cleft in the study group were significantly different (*p* < 0.05), with a higher percentage of surfaces that bled (42%) on probing compared to the upper incisors (IU) of the control group (29%). Children with CLP had a 20% of teeth mobility score of 3.	Compared to controls, young people with CLP showed poor oral hygiene and poor periodontal status.
Quirynen M, 2003. Belgium [25]	Analytical cross section	8-18	75 Bilateral CLP with orthodontic treatment	----	Microbiological culture CFU/mL Saliva, dental plaque and teeth samples	• *Prevotella intermedia* (58.7% vs. 38.7%), *Peptostreptococcus micros* (24% vs. 16%), and *Campylobacter* rectus (56% vs. 46.6%) were slightly higher but not significantly different for neighboring cleavage sites than for contralateral opponents. • There was a significant difference in the median plaque index (1.03) between the sites neighboring the cleft, the contralateral tooth and the teeth neighboring the cleft (*p* = 0.01). • Teeth near or neighboring the cleft had a significantly (*p* < 0.01) greater probing depth and greater losses > 2 mm than their contralaterals.	The teeth adjacent to the unilateral cleft of the lip and palate were not necessarily predisposed to attachment loss.
Da Silva J, 2018. Brazil [26]	Prospective longitudinal descriptive	0-12	46 orofacial clefts indicated for surgical rehabilitation	-------	Fungi cultivation Identification of *Candida* species Presurgical samples of the orofacial and postsurgical fissure of the oral cavity	• Before asepsis in the operating room, they observed oral colonization of candida species in 18 patients (39.1%): *Candida albicans* (15.2%; ≥ or <350 cfu/mL), *Candida tropicalis* (17, 4%; ≥ or <350 cfu/mL) and *Candida krusei* (8.7%; ≥ or <350 cfu/mL) • *Candida* spp*.* frequency of 39.1%, with no correlation with the different types of orofacial clefts or surgical history. • A patient with CLP was suspected to have *Candida tropicalis* ≥ 350 cfu/mL.	The anatomical and physiological characteristics of patients with orofacial clefts could influence the frequencies and dynamics of oral colonization of *Candida* spp*.*
Costa B, 2003. Brazil [10]	Cross section	5-6	30 CLP	27	Microbiological cultures CFU/mL Subgingival plaque samples	• *Prevotella nigrescens* (16.67%) was detected in the experimental group and in the control group (11.11%); *Porphyromonas gingivalis* and *Treponema denticola* were not detected. • Mean gingival index in the experimental group (1.82 ± 0.38) was significantly higher (*p* < 0.05) than that in the control group (0.79 ± 0.33). • Children in the experimental and control groups presented moderate plaque index scores (73.33% and 81.48%, respectively) and high prevalences of mild gingivitis (53.33% and 70.37%).	Children with cleft showed greater gingival inflammation and prevalence of pathogenic microorganisms.
Thomas G, 2012. United Kingdom [27]	Prospective longitudinal descriptive (Incidence)	12 months	144 patients:	---	Microbiological cultivation Nasal and oropharyngeal samples	• 47 patients were positive for *Staphylococcus aureus* (21%), *< Streptococcus* B-hemolytic (3%) and *Streptococcus pneumonia, Haemophilus influenzae, Haemophilus parainfluenzae, Streptococcus millerae, Enterococci* spp.*, Coliforms* spp*., Moraxella* spp*.,* and *Isolate diphteroides* spp. • Beta-hemolytic *Streptococcus* was more common in patients with bilateral CLP before surgery (no source data). • No significant difference was detected in the number of patients with a positive microbiological culture preoperatively compared to perioperatively (48% and 50%).	Preoperative microbiota could not be considered as a predictor of the nasal and oropharyngeal flora at the time of surgery.
Hupkens P, 2007. The Netherlands [28]	Before-after intervention	1-12	124 palatal surgery patients	---	Microbiological cultures Nasal samples and oropharyngeal mucosa	• Positive cultures for *Streptococcus* spp*.* presented in combination with cultures with *Staphylococcus aureus.* • 8 patients with wound infection presented 46 *Haemophilus influenzae,* 10 *Staphylococcus aureus,* 30 *Streptococcus pneumoniae,* 8 *Streptococcus* Hemolytic group A*,* 8 *Streptococcus* Hemolytic group B*,* 3 *Streptococcus* Hemolytic group C*, 0 Streptococcus* Hemolytic group G*,* 1 *Klebsiella ozaena,* 4 *Klebsiella pneumoniae, 2 Serratia liquefaciens, 31 Moraxella catarrhalis,* 4 *Pseudomonas* spp*., 9 Escherichia coli, 5 Acinetobacter* spp., 1 *Citrobacter* spp., 3 *Enterobacter cloacae, 1 Xanthomonas maltoph,* 1 *Candida albicans,* and 1 other yeast. • Of the 124 patients, 8 had positive preoperative cultures for group A *Streptococcus*.	The surgical wound represented the entry of microorganisms which invaded the surgical field.
Sundell A, 2015. Norway [29]	Cases and controls	5-10	133 CLP	297	Dentocult® SM-Strip mutans, Dentocult® LB Microbiological evaluation Saliva samples	• Compared with children in the control group, children with CLP presented significantly higher counts of *Lactobacillus* spp*.;* low risk, 81%; moderate risk, 17%; and high risk, 2% (*p* < 0.05). • There was no increase in *Streptococcus mutans* counts in children with CLP. • Probability of being categorized with high caries risk in the CLP group was significantly high (OR = 1.89; 95% CI = 1.25-2.86).	Children with CLP were more likely to be classified at high risk of cavities and high counts of *Lactobacillus* spp.
Machorowska A, 2017. Poland [1]	Cases and controls	Neonates	30 patients, after 37 weeks, with complete unilateral or bilateral cleft lip and palate 25 patients with isolated soft palate cleft.	----	Microbiological cultures Palatal mucosa samples at the margin of the fissure, dorsum of the tongue and palatal mucosa.	• Patients with CLP presented with significantly higher levels of *Streptococcus mitis,* 63.3% (*p* = 0,002); *Streptococcus salivarius,* 26.6% (*p* = 0,022); *Staphylococcus aureus MSSA,* 40% (*p* < 0.001); *Staphylococcus epidermidis,* 33.3% (*p* < 0.001); *Enterobacter cloacae,* 10% (*p* = 0.007); *Klebsiella pneumoniae,* 20% (*p* < 0.001); and *Klebsiella oxytoca,* 16.6% (*p* < 0.001). • After surgery, there was a statistically significant increase in the percentage of *Gemella morbillorum* (24%) (*p* = 0.041). • *Streptococcus salivarius* in the CLP group after surgery was 22 times higher than before surgery, OR = 22 [95% CI, 2.96-16.21]. • Odds ratio for *Staphylococcus aureus MSSA*, OR = 16 [95% CI, 2.12-12.65] and *Klebsiella oxytoca*, OR = 18 [95% CI, 2.40-13.83], was between 16 and 18 times higher after surgery.	The development of the microbiota in children with CLP was accompanied by a significant increase in commensal and potentially pathogenic organisms. Patients with CLP are at increased risk of developing oral infectious

### Risk of bias analysis of the studies included in the meta-analysis

**Fig. 2:**
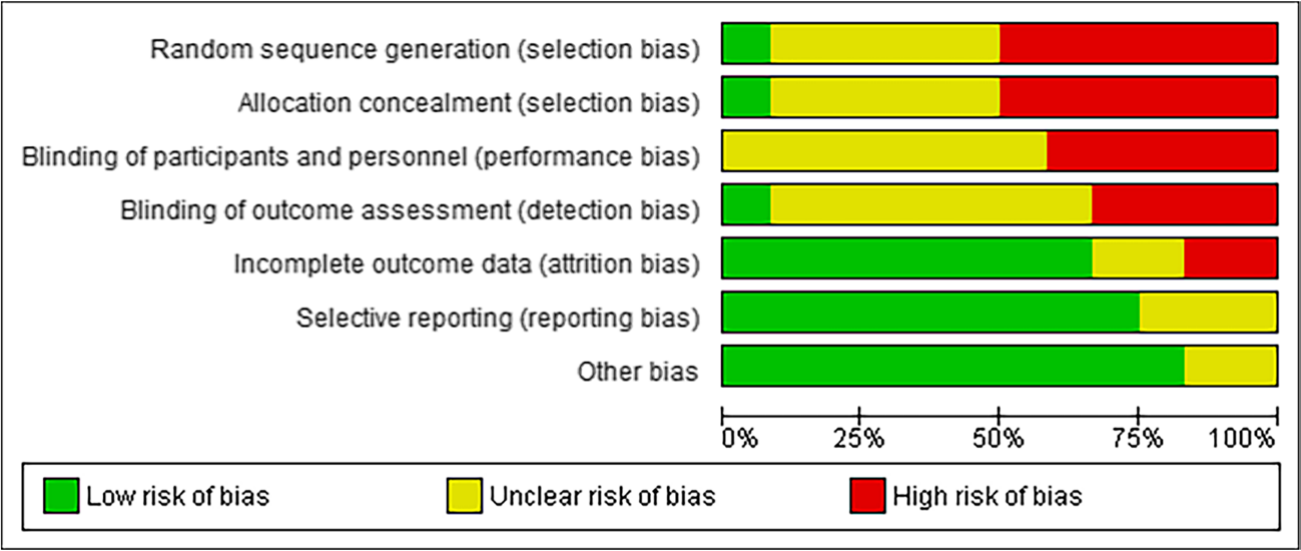
Risk of bias in the studies included in the meta-analysis

Using Rev Manager 5.4, the risk of bias in the included studies was evaluated; the results indicated that the majority of the studies (90%) had a prospective longitudinal case–control design in which there was a high level of bias (Fig. 2).

#### Cariogenic microbiota and its relationship with dental status

Regarding the cariogenic microbiota in lip-palate sites, there are microorganisms similar to those found in patients without clefts, although with differences in the percentage. In studies by Ahluwalia et al. [14], Lucas et al. [15], and Hassani et al. [22], there were significantly higher counts of *Streptococcus mutans* and *Lactobacillus* spp. in patients with CLP. Cheng et al. [20] and Sundell et al. [29] reported significantly higher percentages of *Lactobacillus* spp*.* in patients with this condition. In addition, microorganisms related to endodontic lesions, such as *Peptostreptococcus micros* and *Catonella morbi,* were detected.

Regarding dental status, Ahluwalia et al. [14] reported higher CPOD-ceod index scores (decayed, lost or filled teeth) in CLP patients than in patients in the control group, and Lucas et al. [15] and Durhan et al. [16] did not find statistically significant DMFT index scores for patients with CLP.

#### Periodontopathogenic microbiota and its relationship with the periodontal state.

A characterization of the pathogenic microbiota of the periodontium in patients with CLP is presented as reported by Rawashdeh et al.8 Costa et al.10 Perdikogianni et al. [11], Ahluwalia et al. [14], Funahashi et al. [21], and Quirynen et al. [25]: *Capnocytophaga gingivalis, Eikenella Corrodens, Wolinella* spp*. Actinomyces* spp *, Campylobacter* spp*, Fusobacterium* spp*, Fusobacterium nucleatum, Prevotella intermedia/nigrescens, Peptostreptococcus micros* and *Porphyromonas gingivalis.*

Regarding the gingival and periodontal condition, Ahluwalia et al. [14], Rawashdeh et al. [8], Funahashi et al. [21], Perdikogianni et al. [11], Quirynen et al. [25], and Costa et al. [10] reported a higher gingival index, biofilm index, probing depth, bleeding on probing and loss of insertion in patients with CLP, and Lucas et al. [15] did not find significant differences in periodontal indicators between patients with and without CLP.

#### Oral microbiota and its relationship with type of fissure

There is heterogeneity in relation to the microbiota and type of fissure. Rawashdeh et al. [8], who analyzed of samples of tongue, nasal and palatal mucosa, reported a colonization rate by *Candida* spp*.* that was higher in patients with bilateral cleft lip and palate (77.7%) than in patients with unilateral cleft lip and palate (57, 1%), a finding that was attributed to the fact that patients with clefts have poor oral hygiene.

Bokhout et al. [24], who analyzed saliva and dental plaque microbiological cultures, reported that patients with bilateral cleft lip and palate had a higher percentage of microorganisms: *Streptococcus mutans* (in saliva by 57.1% and in teeth by 71.4%) and *Lactobacillus* spp*.* (in saliva and teeth by 14.3%); however, they reported a lower percentage of these microorganisms in isolated labial fissures and isolated cleft palate.

*Streptococcus mitis, Streptococcus salivarius, Staphylococcus aureus MSSA, Staphylococcus epidermidis*, *Enterobacter cloacae, Klebsiella pneumoniae* and *Klebsiella oxytoca* were more predominant in CLP than cleft soft palate.

Zhang et al.^2^who analyzed saliva and nasal samples through microbial genomic DNA and PCR, revealed that the genera *Lautropia* spp*.* and *Bacillus* spp. were less abundant in saliva samples from individuals with a complete cleft palate (*p* = .029).

#### Oral microbiota and its relationship with surgical intervention

Studies have identified microorganisms associated with the presurgical and postsurgical conditions related to cheilorrhaphy and palatorrhaphy. Cocco et al. [17], Tuna et al. [18], Arief et al. [19], Thomas et al. [27], Hupkens et al. [28], and Machorowska et al. [1] identified different percentages of *Staphylococcus aureus* in patients with CLP. Tuna et al. [18] reported that the transmission of this microorganism increases with the size of the postsurgical residual oronasal fistula. Cocco et al. [17], Hupkens et al. [28], and Thomas et al. [27] reported that beta-hemolytic Streptococcus (*Streptococcus pyogenes*) was associated with a high risk of complications such as dehiscence of the surgical wound. Cocco et al. [17], Machorowska et al. [1], and Hupkens et al. [28] isolated the genera *Klebsiella pneumoniae* and *Klebsiella oxytoca*, which were more predominant in the preoperative period and decreased in proportion after surgery.

Thomas et al. [27] and Hupkens et al. [28] isolated *Moraxella catarrhalis* in patients who underwent surgery, and Machorowska et al. [1] and Cocco et al. [17] isolated methicillin-resistant *Staphylococcus aureus,* which increased in number after surgical repair. Rawashdeh et al. [8] and Da Silva et al. [26] found *Candida* spp., *Candida albicans, Candida krusei* and *Candida tropicalis* before and after surgical repair, reporting higher proportions of this microorganism after surgical intervention, with statistically significant differences, noting that the greater was the number of surgical interventions, the greater the colonization by *Candida* spp.

Liu et al. [9] found significant variations in the microbiota in patients undergoing surgery who presented inflammation compared to those without inflammation, including the following: operational taxonomic units (OTUs) related to inflammation – *Tannerella* spp., *Porphyromonas* spp*., Gemella* spp*., Moraxella* spp*., Prevotella nigrescens* and *Prevotella intermedia*; and related OTUs without the presence of inflammation – *Lautropia* spp., *Neisseria* spp., *Capnocytophaga* spp., *Veillonella dispar, Veillonella parvula* and *Prevotella melaninogenica.* OTUs corresponding to *Streptococcus* spp. and *Prevotella* spp. were present in both groups.

### Quantitative analysis

#### Results of the meta-analysis

A meta-analysis was performed to determine the association between the microorganisms analyzed and dental caries. Of the 23 articles included in this review, 5 were meta-analyzed.

#### Streptococcus mutans

**Fig. 3:**
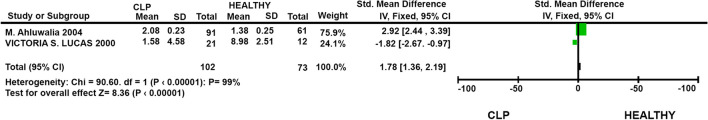
Difference in means in the presence of *Streptococcus mutans*

Two studies were analyzed that reported the outcome in means of the presence of *Streptococcus mutans*, and that there was no difference between the means for healthy patients and those with CLP. The results of these studies show very high heterogeneity, suggesting this analysis should be carefully reviewed (Fig. 3).

#### Lactobacillus

**Fig. 4:**
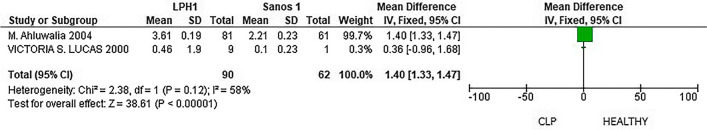
Difference in means in the presence of *Lactobacillus* spp.

For the outcome of the presence of *Lactobacillus* spp., there were no significant differences between patients with CLP and healthy patients, and the diversity of the studies led to high heterogeneity; however, the p value was significant (Fig. 4).

### Caries risk

**Fig. 5:**
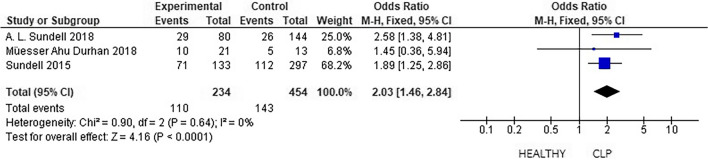
Caries risk assessment

For the analysis of caries risk in patients with CLP and healthy patients, 3 studies were included that evaluated the proportion of patients with caries in both groups. With the 3 included studies, the OR was 2.03, indicating that patients with CLP were 2.03 times more likely to have cavities than were patients in the control group. Heterogeneity was low (*p* < 0.005) (Fig. 5).

## Discussion

Children with CLP have a very diverse microbiome and it has now been reported that polymicrobial communities induce a dysregulated and destructive host response through a global mechanism termed polymicrobial synergy and dysbiosis. Microorganisms in the communities tend to interact synergistically to enhance colonization, persistence or pathogenicity [30].

Regarding the cariogenic microbiota in CLP sites, the results of this investigation showed that there are microorganisms similar to those found in healthy patients, although with a higher percentage in fissure sites, especially higher counts of *Streptococcus mutans* and *Lactobacillus* spp*.* These findings are like those reported by Chaudhari et al. [31], who evaluated the *Streptococcus* and *Lactobacillus* spp*.* Counts in saliva and reported higher counts in patients with cleft as well as increased salivary *Lactobacillus* spp. counts that were higher (60%) in children with CLP. Similar results were reported by Parapanisiou et al. [32], who reported elevated levels of *Lactobacillus* spp*.* (> 10 ^5^ CFU/ml) in patients with CLP.

Contrary to the findings reported in the present investigation, Shashni et al.^7^found no statistically significant difference in terms of *Lactobacillus* spp*.* levels among children with CLP, children with a high risk of caries without cleft and children without cavities and without cleft. Likewise, Dahllöf et al. [33] did not observe differences in the salivary count of *Lactobacillus* spp. in groups with cleft lip and cleft palate.

The higher percentage of *Streptococcus* spp*.* and *Lactobacillus* spp*.* could be related to the anatomical and treatment conditions that patients with CLP present. Shelton et al. [34] reported that presurgical orthopedic devices alter the conditions of the oral cavity, establishing acidic environments as a result. The acrylic material of the plate generally presents roughness, which increases the probability of colonization of *Lactobacillus* spp. and consequently increases *Streptococcus* spp. [34].

In relation to the dental condition, DMFT-ceod index scores were higher for these patients than for the control group. Likewise, patients with CLP had a 2.03× greater probability of presenting caries than did the control group (*p* < 0.005). These findings do not reveal a direct relationship with dental caries; however, the microbiota is an essential biological factor for the development of lesions, its composition is dynamic, and its evolution depends greatly on the intake of sugars and the use of fluoride [18, 20, 22]

These results are similar to those reported by Chaudhari et al. [31], who analyzed the presence of dental caries in patients with and without CLP, reporting an increase in the number of teeth with caries (DMFT scores that increased from 2-3 to 4-6), which was significantly correlated with counts of salivary *Lactobacillus* spp. both in children with CLP and in non-cleft children; there was no significant correlation with counts of salivary *Streptococcus* spp. in children with and without CLP.

Worth et al. [35], in a systematic review and meta-analysis, reported that the overall pooled mean difference in the ceod was 0.63 (95% CI: 0.47 to 0.79) and in the DMFT was 0.28 (95% CI: 0.22 to 0.34), suggesting that individuals with cleft lip and/or palate have a higher frequency of dental caries in both primary and permanent teeth. Contrary to our results, Bastos et al. [36], in a Brazilian population, found no significant differences in dental condition between children with and without CLP.

Several factors could influence the risk of caries in patients with CLP. Allam et al. [37] analyzed primary and mixed dentition in patients with CLP and found a direct correlation between caries, the intake of foods containing sugar between meals and hygiene habits. They also reported that there was a direct correlation between CPOD-ceod index scores and a higher intake of sugary foods.

Regarding the periodontal microbiota, *Campylobacter* spp., *Fusobacterium* spp., *Fusobacterium nucleatum, Prevotella intermedia/nigrescens, Parvimonas micra* and *Porphyromonas gingivalis* have been isolated in patients with CLP and are considered microorganisms of great pathogenic capacity [8, 10, 11, 14, 15, 25] The presence of periodontopathogenic bacteria such as *Porphyromonas gingivalis* is relevant*,* an etiological agent in severe forms of periodontitis, which is not a common disease in patients under 18 years of age; its presence in children and adolescents has been related to immunological alterations as a modification in neutrophil chemotaxis. *Porphyromonas gingivalis* can locally invade periodontal tissues and evade host defense mechanisms. In doing so, it uses virulence factors that cause the dysregulation of innate immune and inflammatory responses [38].

Lamont et al [39] reported that in periodontal diseases, polymicrobial communities induce a dysregulated and destructive host response through a global mechanism termed polymicrobial synergy and dysbiosis. In contrary to what occurs in the gastrointestinal tract, periodontal diseases are associated with an increase in microbiome diversity, which is thought to be a consequence of additional nutrients derived from host tissue damage and increased physical space as the gingival cleft deepens.

Mombelli et al. [40] analyzed the microbiota in patients with unilateral and bilateral CLP and observed the presence of gram-negative anaerobic microorganisms. They also reported the presence of *Fusobacterium* spp., *Prevotella melaninogenica* and *Prevotella intermedia* in patients with CLP, but they did not detect *Porphyromonas* gingivalis, *Actinobacillus* spp. and *Aggregatibacter actinomycetemcomitans* in the study population.

Weckwerth et al. [41] conducted a study with 31 patients with CLP and chronic suppurative otitis media and obtained positive cultures from 83% of the patients. *Pseudomonas aeruginosa* (54.9%)*, Staphylococcus aureus* (25.9%) and *Enterococcus faecalis* (19.2%) were isolated, but no anaerobes were isolated by culture, and the polymerase chain reaction assays revealed 1 or more bacteria in 97.1% of the samples. Anaerobic microorganisms were detected by polymerase chain reaction assays, for example, *Fusobacterium nucleatum, Bacteroides fragilis* and *Peptostreptococcus anaerobius.* This finding suggests that patients with this condition present communication between the ear and the oral cavity, and through this route, there could be an exchange of microorganisms.

In this review, 6 studies identified that patients with CLP presented higher gingival index values and biofilm index values, deeper probing depth, and more bleeding on probing and attachment loss. These results support the results reported by Parapanisiou et al. [32], who found that the biofilm index was significantly higher in patients with CLP than in the control group (*p* = 0.0003). Likewise, Veiga et al [42], in a study with 156 children between 5 and 18 years of age with CLP, showed that fissured patients presented a higher plaque index and gingival index and greater depth when probing.

Plakwicz et al. [43] evaluated the periodontal index in 34 patients with a divided mouth, reporting that the depth of probing and the loss of clinical attachment were greater in the lateral incisors and canines adjacent to the cleft lip than in the same contralateral teeth without the fissure. Wyrębek et al. [44] analyzed 15 patients aged 6 to 18 years with bilateral clefts and found greater bleeding on probing and loss of attachment in the teeth adjacent to the cleft.

The publications analyzed in this study identified microorganisms associated with the pre- and postsurgical conditions related to cheilorrhaphy and palatorrhaphy. Studies had reported a significantly higher count of *Staphylococcus aureus* in saliva samples from children with larger oronasal fistulae and indicated a positive correlation between the size of the fistula and the frequency of transmission of *Staphylococcus aureus* to an oral environment. Adeyemo et al. [45] reported that *Staphylococcus* spp. is a commensal of the skin and nose and that cleft surgery involves both extraoral and intraoral incisions that often lead to communication between the skin and nasal mucosa. Therefore, contamination of the surgical wound in patients with CLP and the subsequent entry of this microorganism into the bloodstream may explain the high prevalence of *Staphylococcus* spp*.* observed in this study. Authors such as Chuo and Timmons [46] conclude that children with unrepaired CLP have an increased risk of carrying *Staphylococcus aureus* and that these risks should be taken into account when choosing the relevant preoperative and postoperative bacteriology tests.

The oral microbiota is highly diverse, consisting of hundreds of bacterial species in the different oral microenvironments, and they play an important role in determining the state of health or disease in the host [47–49]. However, due to their high complexity and the limitations of the methodological tools available to describe the microorganisms associated with health or disease, together with the fact that more than one third of oral bacteria are not culturable, traditional microbiological approaches provide incomplete information on the natural communities that inhabit the oral cavity [50, 51].

In this systematic literature review and meta-analysis, it was observed that of the 23 articles included, 14 (61%) performed microbiological cultures to search for bacteria and 2 (8.7%) of the studies for the identification of fungi, specifically Candida species, 3 (13%) used commercial techniques with pre-established panels of microorganisms such as the CRT test system and Dentocult®, Likewise, 2 (8.7%) performed molecular biology assays, one of them DNA-DNA hybridization and the other PCR-DGGE in which a limited number of bacterial species are analyzed and only 2 (8.7%) of the studies included new generation sequencing techniques or NGS, by amplification of a fragment of 16S rRNA.

PCR amplification of fragments of the gene encoding the 16S rRNA and subsequent sequencing permits the identification of bacterial genera and in some cases even allows the determination of species, depending on the bacterial genus evaluated and the variable region analyzed. This gene has an approximate size of 1500 nucleotides and has 9 variable regions. In studies of microbiota associated to human infectious diseases, the most used region for its analysis is V3-V4 [52].

Recently, sequencing of the entire microbiome including bacteria, archaea, fungi, parasites and DNA-type viruses is being used in different pathologies of medical importance by sequencing all the DNA present in the sample; however, the bioinformatic analysis of all this information obtained is extremely laborious, but it allows us to describe species, subspecies or even strains present, in addition to providing information on the abundance in which they are present. However, to date, it has not been possible to find metagenomic studies that describe the different microbiological profiles in patients with and without NSCLP. Researchers are even proposing to integrate information derived from the metagenome and metatranscriptome simultaneously to truly understand the role of the microbiota in disease generation. The metagenome describes qualitatively and quantitatively the totality of bacteria present in different niches, including those of the oral cavity. The metatranscriptome, on the other hand, provides a profile of bacterial transcripts also qualitatively and quantitatively reflecting the transcriptional activity of all the microorganisms present in the niches under study. These transcripts could reflect the presence of bacterial metabolites.

It is important to mention that the type of sample makes it difficult to compare the studies, since the bacterial community of the subgingival and supragingival microbiota, although they tend to be similar, is different from that found in the mucosa and that found in saliva; in fact, some oral bacteria show a specific tropism towards the different biological surfaces in the oral cavity [53]. For example, Streptococcus salivarius, S. oralis, S. constellatus, S. mitis, S. intermedius and S. anginosus are preferentially found in soft tissues and saliva, compared to Streptococcus sanguis which preferentially colonizes dental surfaces in particular supragingival plaque.

## Conclusions

- The present investigation identified that patients with CLP had higher counts of *Streptococcus mutans* and *Lactobacillus* spp.
- The results of the meta-analysis suggest that individuals with CLP may have greater risk of developing dental caries; therefore, this risk should be taken into account when making clinical decisions and adopting preventive measures to reduce oral comorbidities in these patients.
- Periodontopathogenic bacteria were observed in fissure areas, highlighting the presence of *Porphyromonas gingivalis.*
- The sites adjacent to a fissure have higher plaque index and gingival index values, a deeper probing depth and greater loss of attachment.
- The larger the oronasal fistula is, the greater the percentage of *Staphylococcus* spp.

## Recommendations

The description of the oral microbiota should be carefully interpreted as it is a function of the methodology used, mainly because traditional microbiological cultures present limitations as they only describe that microbiota that can be cultured. We recommend that future studies incorporate a single sample collection method or unify the type of sample. In addition, large-scale clinical studies should be conducted. Metagenomics and metatranscriptomics studies are recommended in children and adolescents with and without non-syndromic cleft lip and palate.

## Limitations

Future studies should incorporate the units of measure for microorganisms and adequately describe their different methods of sample collection to unify knowledge of this topic. Additionally, large-scale clinical studies should be conducted.

## Data Availability

The datasets and studies used and/or analyzed during the current study are available from the corresponding author on reasonable request.

## Abbreviations

CLP: Cleft lip and palate
NSCLP: Nonsyndromic cleft lip and palate
WHO: World Health Organization
ENSAB: National study of oral health
SSP: Species
